# Application of photoacoustic imaging in tumor diagnosis and surgical treatment

**DOI:** 10.3389/fonc.2026.1787591

**Published:** 2026-08-20

**Authors:** Yuwei Dong, Cuirong Li, Xiaoqin Qian

**Affiliations:** 1 Jiangsu University, Zhenjiang, China; 2 Ultrasound Medicine Department, Yunyang People’s Hospital, Danyang, Zhenjiang, China; 3 Ultrasound Medicine Department, Northern Jiangsu People’s Hospital, Yangzhou, China; 4 Yangzhou University Affiliated Subei People's Hospital, Yangzhou, China

**Keywords:** clinical application, NIR-I, NIR-II, photoacoustic imaging, surgery, tumor diagnosis, ultraviolet, vascular imaging

## Abstract

Photoacoustic imaging (PAI) is an emerging hybrid modality that combines high-contrast optical imaging with high-resolution ultrasound imaging, offering significant potential for tumor diagnosis and surgical guidance. This review provides a concise and focused overview of the principles, technological advancements, and clinical applications of PAI in oncology. We begin by introducing the basic mechanism of PAI, including the photoacoustic effect and the differentiation between the NIR-I and NIR-II imaging windows. The review then systematically examines the role of PAI in preoperative tumor characterization, intraoperative margin assessment, and vascular involvement detection. Emphasis is placed on how PAI enables label-free, real-time visualization of tumor microenvironments, including hemoglobin distribution, oxygen saturation, and nuclear morphology. Key technological innovations, such as multispectral optoacoustic tomography (MSOT) and optical-resolution photoacoustic microscopy (OR-PAM), are discussed with a focus on their inherent depth-resolution trade-offs. We also highlight recent advances in exogenous contrast agents and their potential for molecular imaging. critically evaluating their stability, toxicity, and translational hurdles. By synthesizing current evidence and directly addressing key technical and clinical limitations—including penetration depth, laser safety, reconstruction artifacts, and lack of standardization—this review provides a comprehensive and critical evaluation of PAI’s capabilities, limitations, and future directions in tumor diagnosis and surgical treatment.

## 1 Introduction

Cancer remains one of the leading causes of morbidity and mortality worldwide, with approximately 14 million new cases and 8 million deaths annually (1). Conventional optical imaging techniques, such as optical coherence tomography and multiphoton microscopy, are limited by light scattering, resulting in shallow penetration depths (typically millimeter-scale) (2). In contrast, ultrasound imaging offers deeper tissue penetration due to lower scattering but suffers from poor contrast because image formation relies on acoustic impedance differences rather than biochemical properties (3). Photoacoustic imaging (PAI) has emerged as a powerful hybrid modality that overcomes these limitations by combining optical contrast with ultrasonic resolution (4). However, to appreciate its true potential and limitations, a mechanistic understanding of its signal chain is essential.

The photoacoustic effect involves the absorption of short-pulsed laser light by endogenous chromophores (e.g., hemoglobin, melanin, lipids) or exogenous contrast agents. This absorption leads to transient thermoelastic expansion and the generation of broadband (MHz to tens of MHz) acoustic waves. Critically, the initial pressure rise (p0) is governed by p0 = Γ·μa·F, where Γ is the Grüneisen parameter (a dimensionless constant describing thermoelastic efficiency), μa is the absorption coefficient (cm^-^¹), and F is the local laser fluence (J/cm²) (4, 5). This equation highlights that PA signal strength is linearly proportional to μa, providing the basis for quantitative imaging, but also introduces a key trade-off: increasing F to enhance signal depth carries the risk of exceeding the maximum permissible exposure (MPE) limits set by safety standards (e.g., ANSI Z136.1), potentially causing thermal damage (6). These waves are detected by ultrasound transducers and used to reconstruct images that map the distribution of optical absorption (4). The relationship between tissue penetration depth and laser wavelength is governed by optical scattering and absorption coefficients, which can be mathematically described by the diffusion equation and the Beer-Lambert law. Specifically, the penetration depth δ is approximately given by δ ≈ 1/√(3μ_aμ_s’), where μ_a is the absorption coefficient and μ_s’ is the reduced scattering coefficient. Longer wavelengths in the NIR-II region (1000–1700 nm) experience reduced scattering, enabling deeper imaging (7, 8). For instance, Bag and Ghorai (2015) systematically investigated the ligand field effect to improve photoacoustic properties of organometallic carbonyl clusters, demonstrating the importance of wavelength selection in optimizing PA signal generation (9).

PAI can be classified by its excitation wavelength, as different spectral regions offer distinct advantages for imaging depth, resolution, and molecular specificity. The ultraviolet (UV) and visible ranges enable high-resolution imaging of superficial structures, such as cell nuclei, while the first near-infrared (NIR-I, 650–1000 nm) and second near-infrared (NIR-II, 1000–1700 nm) windows provide deeper tissue penetration for clinical applications (10). This review aims to provide a critical evaluation of PAI’s role in tumor diagnosis and surgical treatment, with a focus on its technical principles, clinical applications, and future directions. Unlike previous reviews, we systematically analyze the fundamental trade-offs—particularly between depth, resolution, and safety—mechanistically link wavelength-dependent properties to specific surgical outcomes, and offer a dedicated, in-depth discussion of the technical, biological, and regulatory hurdles that currently impede widespread clinical translation. The historical development of the field is outlined, highlighting key contributions from leading research groups such as those of Wang, Razansky, and Ntziachristos (11–13).

## 2 Photoacoustic imaging across the spectrum: from ultraviolet to near-infrared

**Figure 1:**
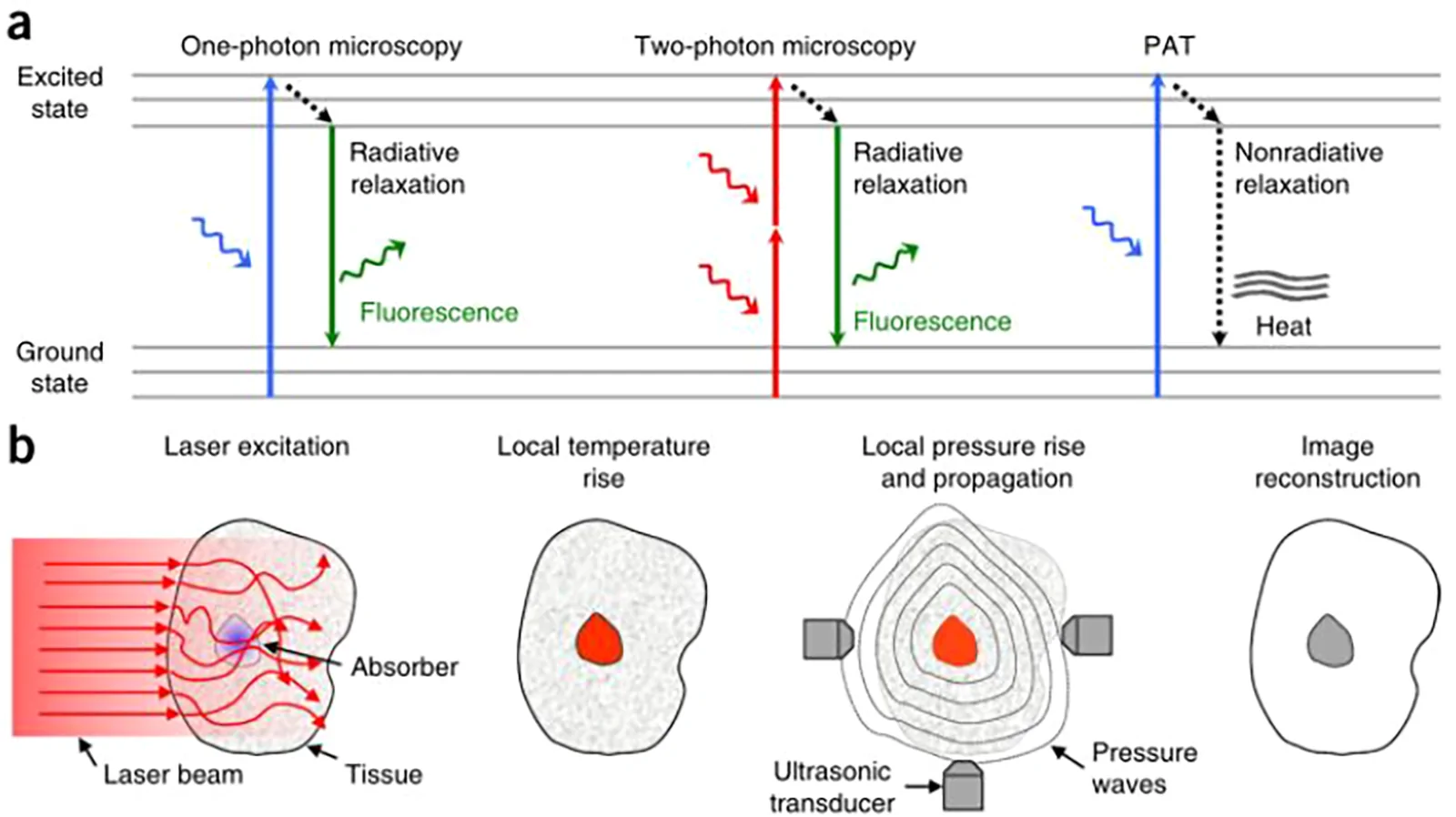
**(a)** Jablonski diagram, illustrating the photon energy transfer in one-photon fluorescence microscopy, two-photon fluorescence microscopy, and PAT. The most common electronic absorption in the visible and ultraviolet spectral region is shown. **(b)** Imaging principle of PAT (4).

Photoacoustic imaging is not defined by a single wavelength but rather encompasses a broad spectral range from ultraviolet (UV) to the near-infrared (NIR). The choice of excitation wavelength is a critical determinant of imaging performance, influencing penetration depth, spatial resolution, and the specific chromophores that can be targeted. This section provides a balanced overview of these spectral windows, with a particular emphasis on the NIR-I and NIR-II windows for deep-tissue imaging, while explicitly positioning UV and visible excitation within the overall framework for high-resolution applications, such as surgical margin assessment (**Figure 1**).

### 2.1 Ultraviolet and visible photoacoustic imaging: high-resolution and label-free histology

At the shortest wavelengths, ultraviolet (UV) and visible light offer the highest resolution due to minimal optical diffraction and strong absorption by endogenous chromophores. UV-PAI, typically using wavelengths around 266 nm, exploits the strong absorption of DNA and RNA in cell nuclei (14). This enables “virtual histology,” generating images that closely resemble hematoxylin and eosin (H&E)-stained sections without the need for tissue processing, making it ideal for intraoperative margin assessment. Visible wavelengths, such as 532 nm, are strongly absorbed by hemoglobin, providing label-free, high-resolution imaging of microvascular networks. These modalities, however, are limited to superficial depths (typically <1 mm) due to strong optical scattering (15).

### 2.2 Near-infrared window I: the clinical workhorse

The NIR-I window (650–1000 nm) is the most commonly used spectral range for clinical PAI due to the availability of commercial lasers and contrast agents. Endogenous chromophores such as hemoglobin, melanin, and lipids exhibit strong absorption in this region, enabling label-free imaging of vascular structures and pigmented lesions (16). Exogenous contrast agents, including indocyanine green (ICG), methylene blue, and gold nanoparticles, have been extensively developed to enhance sensitivity and enable molecular targeting (1). For example, Gao et al. (2008) used 780 nm NIR-I excitation to image breast cancer, demonstrating that intraductal papillomatosis and localized canceration could be visualized in accordance with X-ray findings (17).

However, biological tissues still exhibit significant light scattering and absorption in the NIR-I region, which limits penetration depth and imaging contrast (7). This has motivated the exploration of longer wavelengths in the NIR-II window.

### 2.3 Near-infrared window II: deeper penetration for deep-seated tumors

**Figure 2:**
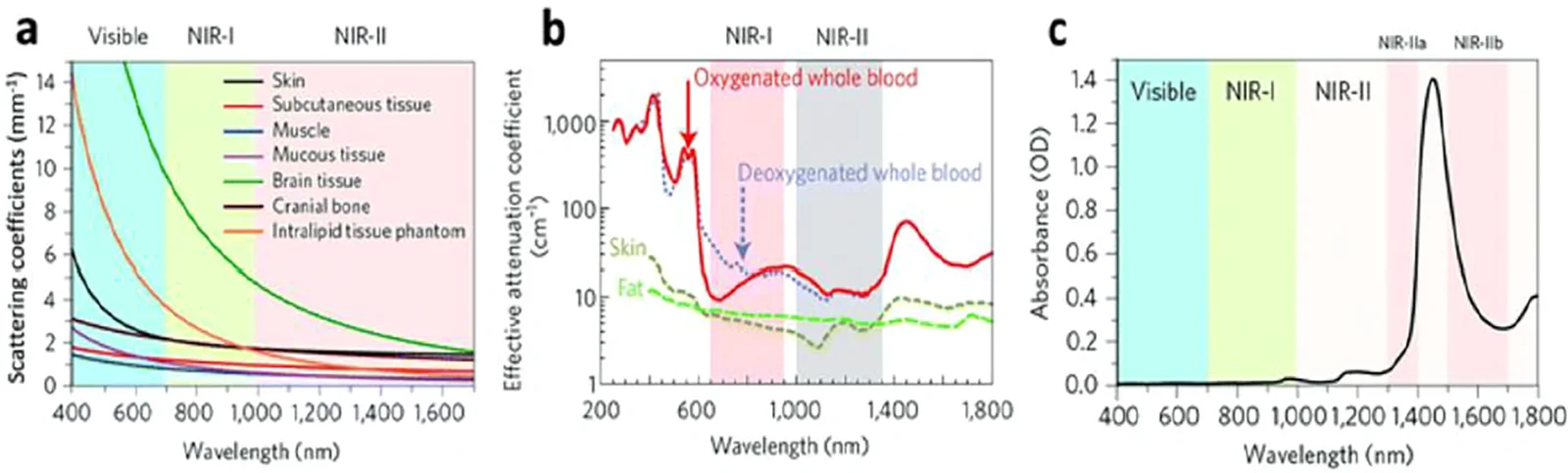
**(a)** The light scattering coefficients of different biological tissues in the range of 400–1700 nm; **(b)** The effective attenuation coefficient of blood, skin and fat to light in the range of 200–1800 nm; **(c)** Absorption spectra of water (15).

The NIR-II window (1000–1700 nm) offers several advantages over NIR-I, including reduced light scattering and lower background absorption from hemoglobin and melanin, leading to deeper tissue penetration and higher contrast (18). As shown in **Figure 2a**, the scattering coefficient of biological tissues decreases monotonically with increasing wavelength, while absorption by water becomes significant only above 1400 nm (**Figure 2c**) (8). Therefore, most NIR-II PAI studies employ wavelengths in the 1000–1400 nm range to avoid water absorption.

[A wide variety of contrast agents have been developed for NIR-II PAI, including organic materials (e.g., conjugated polymers, semiconductor polymers), inorganic nanomaterials (e.g., metal sulfides, carbon nanotubes, MXenes), and hybrid organic–inorganic systems (10). These agents have been applied to image tumors, cerebrovascular networks, and inflammatory processes, as well as to monitor therapeutic responses (19). For instance, Zhao (2024) developed a metal–organic framework (MOF) nanoprobe that responds to hydrogen sulfide (H2S) in colon cancer, enabling ratiometric photoacoustic imaging at 950 nm and 1280 nm to monitor drug release and therapeutic efficacy (19). This example illustrates the potential of NIR-II PAI for theranostic applications.

### 2.4 Mechanistic link between wavelength and clinical outcome

The choice of spectral window is not merely a technical preference but has direct implications for specific clinical tasks. For superficial tumor margin assessment (e.g., melanoma, oral cancer), where penetration depth of a few millimeters is sufficient, the strong absorption of endogenous chromophores in the UV/visible and NIR-I windows (e.g., melanin at 680–750 nm, hemoglobin at 532 nm, DNA at 266 nm) provides excellent contrast and high resolution, enabling label-free “virtual histology” (14, 20). In contrast, for preoperative imaging of deep-seated tumors (e.g., breast, prostate, liver), where the target lies several centimeters below the skin, the reduced scattering of the NIR-II window is critical to deliver sufficient photons to the target and recover acoustic waves with an acceptable signal-to-noise ratio (SNR) (10, 21). Furthermore, molecular imaging of specific biomarkers can benefit from the NIR-II window by using targeted contrast agents (e.g., MOFs, conjugated polymers) that absorb in this range, effectively eliminating background autofluorescence and hemoglobin absorption, thereby providing a “molecular dark field” for high specificity (19). Therefore, the optimal imaging window is a function of tumor depth, desired resolution, and the specific diagnostic or surgical question being asked.

## 3 The application of photoacoustic imaging in tumor diagnosis and operative treatment

### 3.1 Preoperative diagnosis

#### 3.1.1 Structural imaging

PAI enables high-resolution structural imaging of tumors by detecting endogenous chromophores such as hemoglobin and melanin. Breathnach et al. (2018) demonstrated that PAI could accurately measure melanoma thickness preoperatively, with strong correlation to postoperative histology (mean absolute error 0.18 mm), suggesting its potential for noninvasive melanoma diagnosis (20). Similarly, Zhou et al. (2017) used a handheld photoacoustic probe to assess melanoma depth in patients, achieving excellent agreement with excised tissue measurements (22). These studies underscore the feasibility of translating PAI into clinical practice for preoperative tumor staging.

In addition to melanoma, PAI has been applied to visualize tumor-associated vasculature. Omar et al. (2015) used raster-scan optoacoustic mesoscopy to monitor tumor growth and angiogenesis in a murine melanoma model, capturing detailed vascular changes over time (23). Lin et al. (2015) employed optical-resolution photoacoustic microscopy (OR-PAM) to longitudinally track vascular curvature, diameter expansion, and increased blood supply in a mouse tumor model (15). These structural insights are critical for surgical planning, as they help identify involved vessels and guide resection strategies.

**Figure 3:**
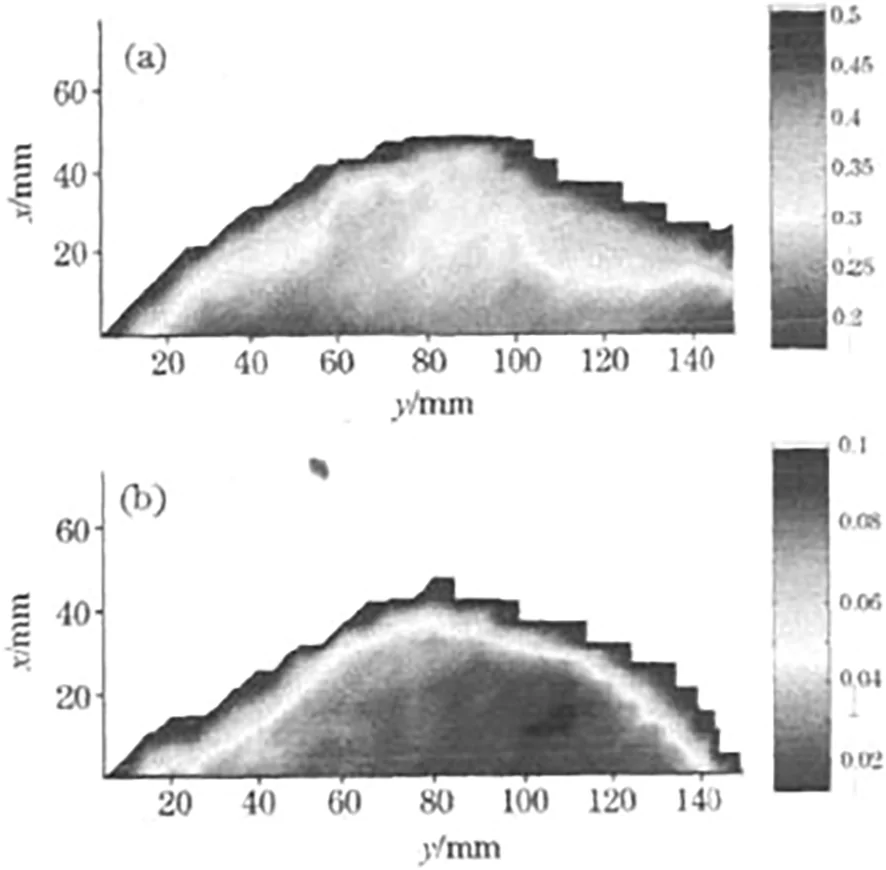
Early diagnostic images of breast cancer using the NIR-I spectral window. **(a)** Normal left breast; **(b)** Right breast cancer.

For breast cancer, early diagnostic images obtained with NIR-I excitation are displayed in **Figure 3** (17).

#### 3.1.2 Functional imaging

**Figure 4:**
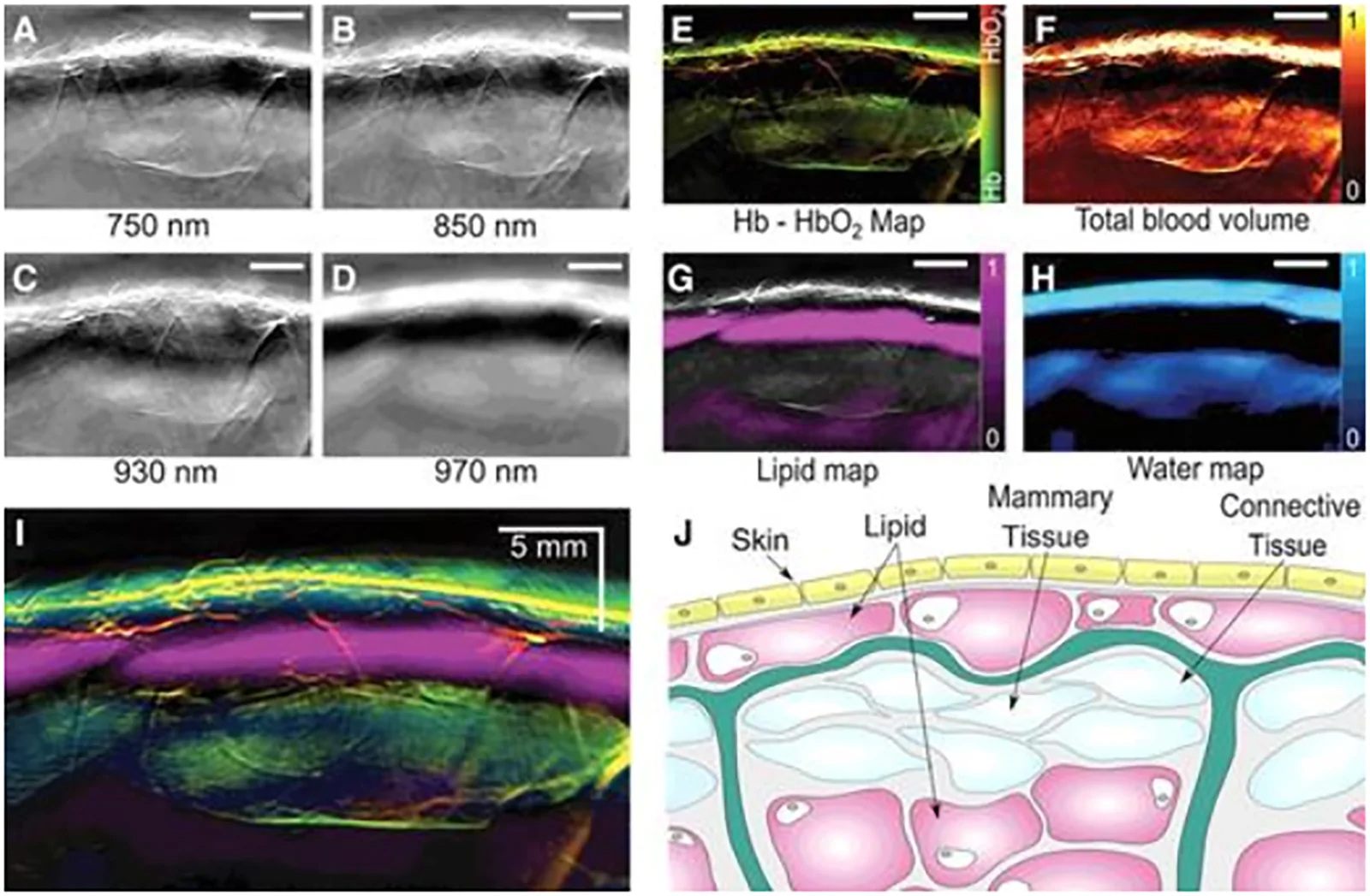
MSOT images of healthy breast tissue revealing spectral and structural information from four endogenous absorbers. **(A–D)**, Raw images acquired at four different wavelengths. **(E)**, Image of HbO2 and Hb distribution after unmixing of the raw images **(A–D)**. **(F)**, Image of total blood volume distribution. **(G, H)**, Lipid and water components after unmixing as in **(E, F)**. **(I)**, Composite image of all four absorbers, revealing the layered structure of the breast. **(J)**, Schematic of the structure and layers of the human breast: yellow, skin; pink, lipid; light blue, mammary tissue; green, connective tissue known as Cooper ligament. Scale bars, 5 mm (25).

Functional PAI provides quantitative maps of hemoglobin oxygen saturation (sO2) and total hemoglobin concentration, reflecting tumor metabolism and hypoxia. Li et al. (2015) developed a functional photoacoustic tomography (fPAT) system capable of distinguishing benign from malignant breast lesions based on vascular oxygenation patterns, with sub-millimeter resolution (24). Diot et al. (2017) used multispectral optoacoustic tomography (MSOT) to image breast cancer patients, revealing increased total blood volume around tumors and invasion of lipid and water layers, which may aid in tumor characterization (21). **Figure 4** exemplifies the multimodal capability of MSOT in visualizing different tissue components, including hemoglobin, lipid, and water (25).

#### 3.1.3 Molecular imaging

Exogenous contrast agents functionalized with targeting ligands enable molecular PAI of cancer-specific biomarkers. Kim et al. (2010) used bioconjugated gold nanocages to target melanomas *in vivo*, achieving strong photoacoustic contrast (26). Li et al. (2008) demonstrated simultaneous multi-target imaging using antibody-conjugated gold nanorods, where plasmon resonance coupling between adjacent nanoparticles altered the absorption spectrum and enhanced PA signal (27). Such approaches hold promise for personalized medicine by visualizing receptor expression and guiding targeted therapies.

#### 3.1.4 Quantitative comparison with established modalities

To contextualize PAI’s role in oncology, a direct comparison with clinical gold standards is essential. MRI offers excellent soft-tissue contrast and depth (whole-body) but has low throughput, high cost, and uses exogenous gadolinium-based agents with associated toxicity risks. CT provides high-resolution anatomical detail and depth rapidly but involves ionizing radiation and poor soft-tissue contrast without iodinated agents. PET is highly sensitive (picomolar) for molecular targets but suffers from poor spatial resolution (4–6 mm), ionizing radiation, and high cost (28). PAI occupies a unique niche: it provides molecular contrast (nanomolar to micromolar sensitivity, depending on the agent) with high spatial resolution (sub-millimeter to micrometer-scale) at depths up to several centimeters, without ionizing radiation. However, its penetration depth is fundamentally inferior to MRI/CT/PET (which have no practical depth limit in clinical systems) (4, 7). Therefore, PAI is not a replacement for whole-body staging but rather a complementary modality for dedicated regional imaging (e.g., breast, thyroid, prostate, surgical bed) where its unique combination of molecular sensitivity, resolution, and real-time capability offers distinct advantages for guiding biopsies or surgical resection (29)s.

### 3.2 Intraoperative guidance: surgical margin assessment and vascular involvement

**Figure 5:**
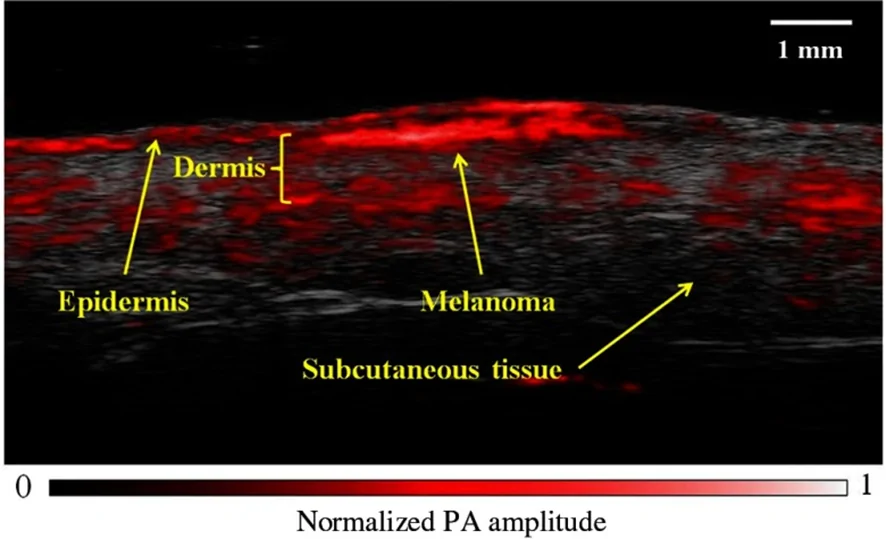
Coregistered PAI image of *in situ* melanoma on upper left extremity. Handheld linear-array based PAI was able to image pigmented lesion and skin architecture with high contrast, speed, and resolution (14).

Accurate assessment of surgical margins is critical to achieving complete tumor resection and reducing local recurrence. Currently, histopathological examination of resected tissues using hematoxylin and eosin (H&E) staining remains the gold standard, but it is time-consuming and unsuitable for intraoperative decision-making (30). PAI offers a label-free alternative for real-time margin assessment (**Figure 5**).

Optical-resolution photoacoustic microscopy (OR-PAM) using a 266 nm laser exploits the strong ultraviolet absorption of DNA and RNA in cell nuclei, generating images that closely resemble H&E-stained sections (14). This technique, known as “virtual histology,” enables rapid intraoperative identification of residual tumor cells at the resection margin without the need for tissue processing. Restall et al. (2021) developed a photoacoustic remote sensing (PARS) system that combines 266 nm excitation for nuclear contrast with optical detection of scattering signals, producing virtual H&E images that correlate well with conventional histology (31). PARS operates without physical contact with the tissue, making it suitable for *in vivo* applications.

In addition to margin assessment, intraoperative visualization of peritumoral blood vessels is essential to ensure complete resection of involved vasculature and reduce postoperative recurrence (32). A dual-wavelength PAI system has been proposed to address this need: 532 nm excitation images hemoglobin in blood vessels, while 266 nm excitation provides nuclear contrast for margin assessment (33). This approach allows surgeons to simultaneously evaluate vascular involvement and margin status during the same procedure, potentially reducing the need for secondary surgeries.

### 3.3 The depth-resolution trade-off: OR-PAM vs. MSOT

A fundamental limitation in PAI, directly addressing Question 3, is the inverse relationship between spatial resolution and imaging depth. This is not a technological shortcoming but a consequence of physics (5, 34). Optical-resolution photoacoustic microscopy (OR-PAM) focuses light to a diffraction-limited spot, achieving micrometer-scale resolution (e.g., <5 µm). However, due to strong light scattering, this high resolution is maintained only within the optical diffusion limit (~1 mm in tissue). OR-PAM is therefore ideal for imaging superficial capillaries, cell nuclei in excised margins, or the tumor surface *in vivo* (14, 15). In contrast, acoustic-resolution (AR)-PAI/MSOT uses unfocused or weakly focused light illumination and relies on the acoustic detection for resolution. While the optical penetration can reach several centimeters, the spatial resolution is determined by the ultrasound transducer’s center frequency and bandwidth. For example, a 5 MHz transducer provides a resolution of a few hundred micrometers, insufficient for visualizing individual cells or capillaries but adequate for visualizing large vessels and tumor boundaries (21, 34). Therefore, the choice of PAI system mandates a deliberate trade-off: select OR-PAM for histology-like detail at the surface and MSOT/AR-PAI for tomographic imaging of deep structures (**Table 1**). Hybrid systems that integrate both modes are an active area of research to address this trade-off.

**Table 1:** Compares the key features of NIR-I and NIR-II photoacoustic imaging.

Feature	NIR-I (650–1000 nm)	NIR-II (1000–1700 nm)
Tissue penetration	Moderate	High
Scattering	Higher	Lower
Background absorption	Moderate (hemoglobin, melanin)	Low (except water >1400 nm)
Typical contrast agents	ICG, MB, AuNRs, QDs	Conjugated polymers, MOFs, CuS, carbon nanotubes
Clinical application	Breast cancer, melanoma	Deep-seated tumors, theranostics

## 4 A critical reassessment of limitations and challenges

While PAI holds great promise, its clinical translation is hindered by several interconnected technical, biological, and regulatory challenges that require a more nuanced discussion than is typically presented.

### 4.1 Physical and technical limitations

Depth-Resolution-Laser Safety Trilemma: As discussed, imaging depth and resolution are inversely linked. Attempting to increase depth by using longer wavelengths and higher laser fluence is constrained by the ANSI MPE limit (e.g., 20 mJ/cm² at 532 nm for a 10 ns pulse) (6). Exceeding this limit risks thermal and mechanical tissue damage. Therefore, for a given imaging depth, there is a maximum achievable SNR, and thus a maximum resolution. Most preclinical studies operate at fluences near the MPE limit, leaving little margin for clinical safety factors (5).

Acoustic Attenuation and Artifacts: The generated broadband PA signal (0.1–100 MHz) is attenuated by tissue at a frequency-dependent rate (~0.5-1.0 dB/cm/MHz). This means higher-frequency components (carrying fine resolution) are preferentially absorbed, blurring the image with depth. Furthermore, acoustic heterogeneities (e.g., bone, air interfaces, fat lobules) cause reflection, refraction, and scattering of the PA waves, leading to reconstruction artifacts (e.g., streaking, shadowing, clutter) that can mimic or obscure tumors. Standard linear reconstruction algorithms (e.g., back-projection) often fail in such heterogeneous media, requiring advanced, computationally expensive models that incorporate sound speed variations (35). Transducer Limitations: Ultrasound transducers are typically designed for either high resolution (high frequency, narrow bandwidth) or deep penetration (low frequency, wide bandwidth). Capturing the full PA signal, which spans two decades of frequency, requires transducers with ultra-wideband response (>100% fractional bandwidth). Current clinical transducers rarely meet this specification, leading to signal loss and image degradation (34).

### 4.2 Exogenous contrast agents: from bench to bedside

The development of targeted molecular contrast agents is a major frontier, but significant hurdles remain (36).

*In Vivo* Signal Generation vs. *In Vitro* Characterization: An agent’s absorption spectrum in a cuvette is a poor predictor of its *in vivo* performance. In the biological environment, agents are subject to protein corona formation, which can alter their size, surface charge, aggregation state, and absorption cross-section, often quenching the PA signal. Furthermore, the local thermoelastic environment (Γ) varies between tissues (e.g., fat vs. muscle), affecting signal strength independently of absorption (36, 37).

Stability and Toxicity Under Laser Excitation: Repeated pulsed laser excitation can heat contrast agents, leading to photodegradation, shape changes (e.g., gold nanorods melting into nanospheres), or explosive vaporization. These processes not only destroy the agent but can also release toxic byproducts (e.g., cytotoxic surfactants from gold nanorods) and cause unintended photothermal damage. The long-term biodistribution and clearance of many nanomaterials (e.g., carbon nanotubes, quantum dots) remain unresolved, with concerns about reticuloendothelial system accumulation and potential chronic toxicity (37).

Theranostic Trade-off: For agents intended for combined photoacoustic imaging and photothermal therapy (PTT), there is an intrinsic conflict. High photo-thermal conversion efficiency (PCE) maximizes tumor heating for therapy but converts absorbed light into heat rather than acoustic waves, reducing PA signal strength. Conversely, a high PA signal requires efficient thermoelastic expansion, which may not maximize heating. Optimizing this ratio for a given application is an active area of research (38).

### 4.3 Clinical translation, standardization, and regulatory barriers lack of standardization

There are no universally accepted phantoms, imaging procedures, or quantification metrics for PAI. This makes it impossible to directly compare results across multiple systems or research groups, severely hindering multi-center trials and regulatory approval (39).

Regulatory Obstacles: The US FDA and European CE mark approval require extensive safety and efficacy data. For PAI systems, this includes not only laser safety (a Class IV laser product) but also the entire ultrasound detection system. For new contrast agents, the pathway is even more arduous, requiring demonstration of safety (toxicology, biodistribution, immunogenicity), Good Manufacturing Practice (GMP) production, and then phased clinical trials. The high cost and long timeline (often >10 years) are major disincentives to commercial development (29, 37).

## 5 Summary and future perspectives: addressing the gaps

In summary, photoacoustic imaging has emerged as a versatile and powerful tool for tumor diagnosis and surgical guidance. Its ability to provide high-resolution, label-free images of structural, functional, and molecular features offers unique advantages over conventional imaging modalities. However, as detailed above, significant limitations must be addressed to facilitate clinical adoption. Future research must shift from descriptive demonstrations to targeted solutions for these well-defined problems. This section directly maps future directions to the limitations identified in Section 4.

### 5.1 Multimodal integration to overcome physical limitations

The depth-resolution trilemma cannot be solved by PAI alone. The most promising path is synergistic integration: (1) PAI-US: Co-registering PAI with clinical ultrasound combines molecular/functional PAI contrast with the deep penetration, real-time B-mode guidance, and established workflow of ultrasound. The US transducer serves dual duty, and US can provide acoustic property maps to correct PAI reconstruction artifacts. (2) PAI-MRI: Combining PAI’s high-resolution molecular imaging with MRI’s whole-field, multi-parametric capability could be transformative for breast and prostate imaging. (3) Optical-PAI Confocal Microscopy: Merging OR-PAM with confocal fluorescence could provide simultaneous nuclear (UV PAI) and molecular (fluorescence) contrast for intraoperative histology[28.29].

### 5.2 AI-powered reconstruction and quantification: machine

learning (ML) offers solutions to many of the challenges outlined: (1) Artifact reduction: Deep learning models (e.g., U-Net, generative adversarial networks) can be trained to map artifact-laden images to clean reconstructions learned from simulations or paired ground-truth data. (2) Speed: ML can enable high-quality image reconstruction from sparse data, reducing scan times and laser exposure. (3) Standardization and Quantification: ML algorithms can correct for system- and operator-dependent variations, enabling absolute quantification of parameters like sO2. (4) Feature extraction: ML-based radiomics can extract hundreds of quantitative features from PAI images to build diagnostic or prognostic models. To achieve this, the field must urgently adopt standardized data formats (e.g., DICOM for PAI) and share open-source datasets for algorithm training (39–41).

### 5.3 Designing clinically translatable contrast agents

Instead of pursuing ever more complex nanomaterials, the field should prioritize agents with: (1) FDA-approved or GRAS (Generally Recognized As Safe) status, such as ICG, methylene blue, or iron oxide nanoparticles (e.g., Ferumoxytol). (2) Clear, rapid clearance pathways (renal or hepatobiliary). (3) High photostability and defined PCE/PA ratio. (4) Simple, scalable GMP synthesis. A shift towards repurposing existing clinical agents or designing “simple but smart” molecularly activated probes will accelerate translation (36–38).

### 5.4 Clinical validation via large-scale, multi-center

To definitively determine whether photoacoustic imaging (PAI) improves patient-relevant outcomes, the field would benefit from moving beyond the current evidence base, which consists primarily of small, single-center feasibility studies. Prospective, multi-center trials are necessary to demonstrate a direct clinical impact, including: (1) a reduction in positive surgical margin rates in breast-conserving surgery using intraoperative PAI guidance compared to standard palpation or visual inspection; (2) improved diagnostic accuracy for lymph node metastasis relative to standard-of-care imaging; and (3) a reduction in the rate of secondary surgeries or local recurrence. Such trials require standardized imaging protocols, validated clinical endpoints, and sufficient statistical power to ensure reproducible and generalizable results (29).

With ongoing technological advances and interdisciplinary collaboration that directly targets the limitations discussed herein, photoacoustic imaging is poised to play an increasingly important role in personalized cancer care, offering real-time, non-invasive, and molecularly sensitive guidance for diagnosis and treatment.
